# Supplementary material for: Structure of the intact tail machine of Anabaena myophage A-1(L)
Source: Nat Commun. 2024 Mar 26;15:2654. doi: 10.1038/s41467-024-47006-z (PMC10966104; doi:10.1038/s41467-024-47006-z)
Supplement: Supplementary file 1 — Supplementary Information [file 41467_2024_47006_MOESM1_ESM.pdf]

Supplementary Information

Figures and figure legends

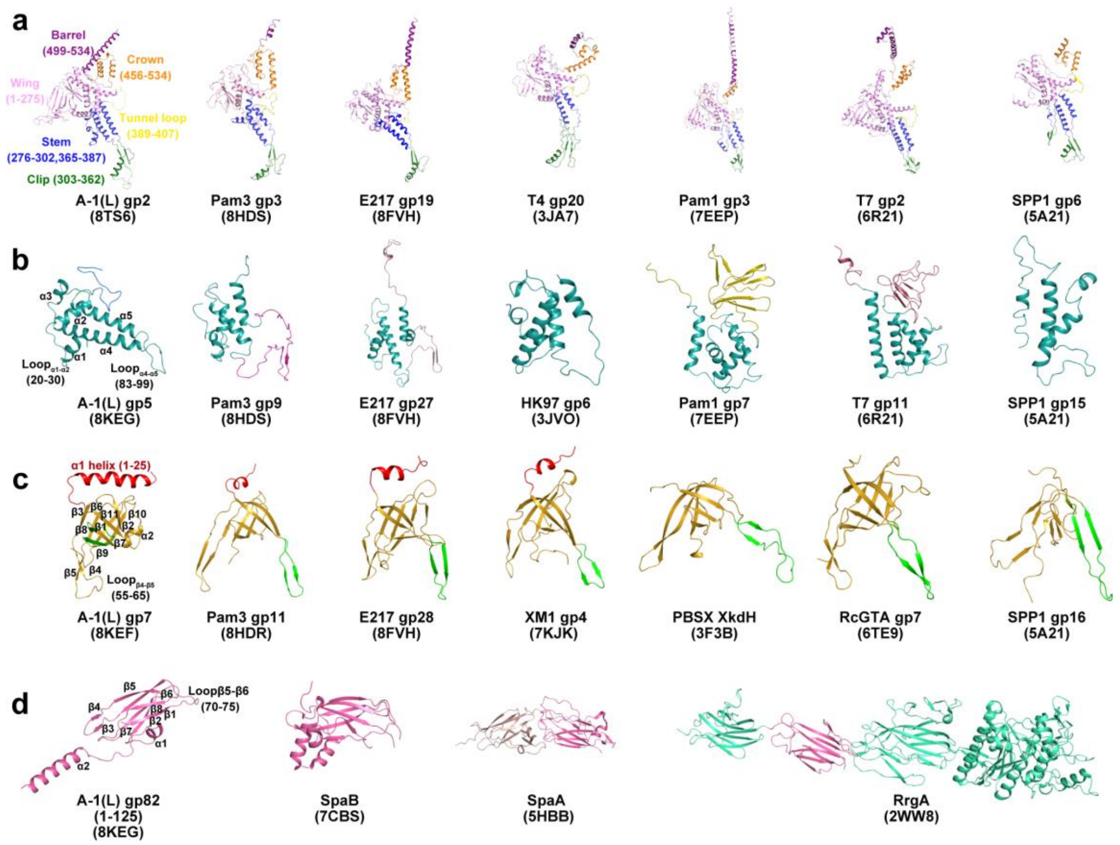

Supplementary Fig. 1 Structures of the neck proteins. a-c, Structures of A-1(L) portal/gp2 (**a**), neck/gp5 (**b**) and neck/gp7 (**c**), in addition to their corresponding homologs from different phages. **d,** Structures of A-1(L) neck fiber/gp82N and its homologs from bacterial pili. The secondary structure elements of A-1(L) neck proteins are labeled.

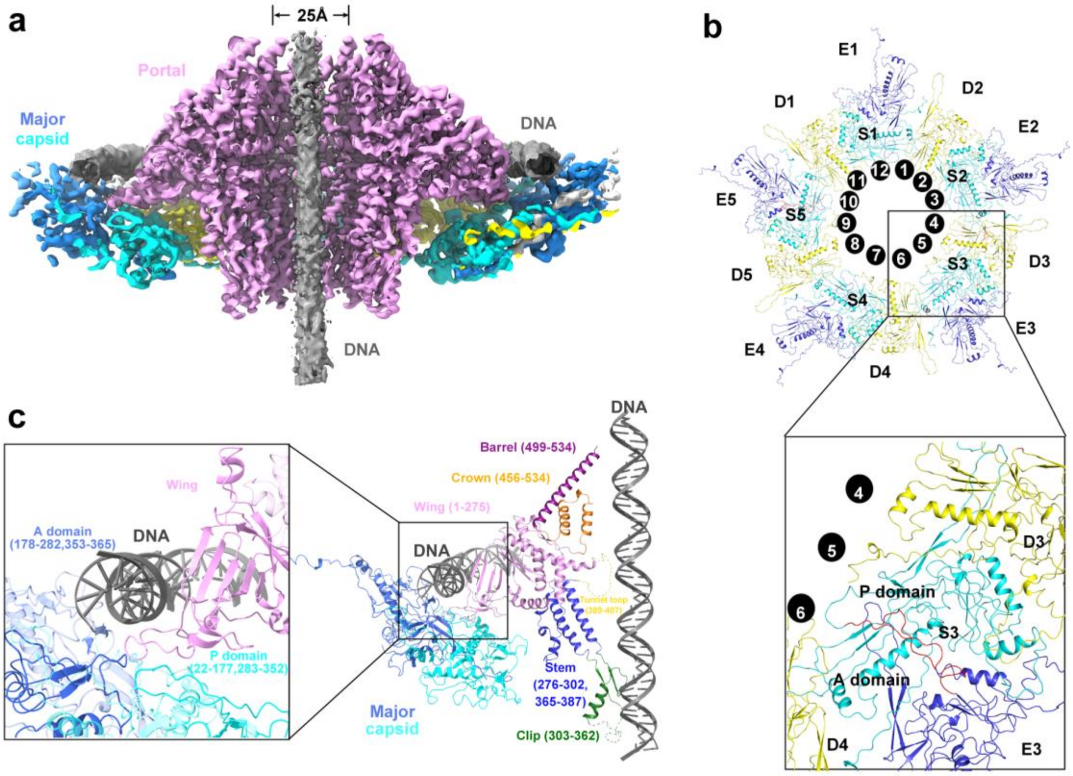

Supplementary Fig. 2 The symmetry-mismatched structure of the 12-fold portal and 5-fold capsid. a, Longitudinal cut view of the portal vertex. The EM densities for DNA, portal and major capsid are colored in dark gray, pink and blue/cyan/yellow, respectively. The diameter of the portal central channel at the narrowest tunnel loop is labeled. **b,** The portal vertex seen from the inside of the capsid. The surrounding major capsid hexamers are shown as cartoons, whereas the 12 subunits of portal are displayed as black circles and sequentially labeled. The surrounding (S1-S5), distal (D1-D5) and external subunits (E1-E5) of the major capsid hexamers are colored in cyan, yellow, and blue, respectively. The inset shows an enlarged view of the interface between the major capsid hexamers and the portal. The secondary structure elements involved in the interaction are colored in red. **c,** Side view of the interface between one portal subunit and two major capsid subunits (blue/cyan). The portal/gp2 subunit is shown as cartoons

with domains colored differentially. The inset shows a circular cleft that accommodates one segment of the genomic dsDNA.

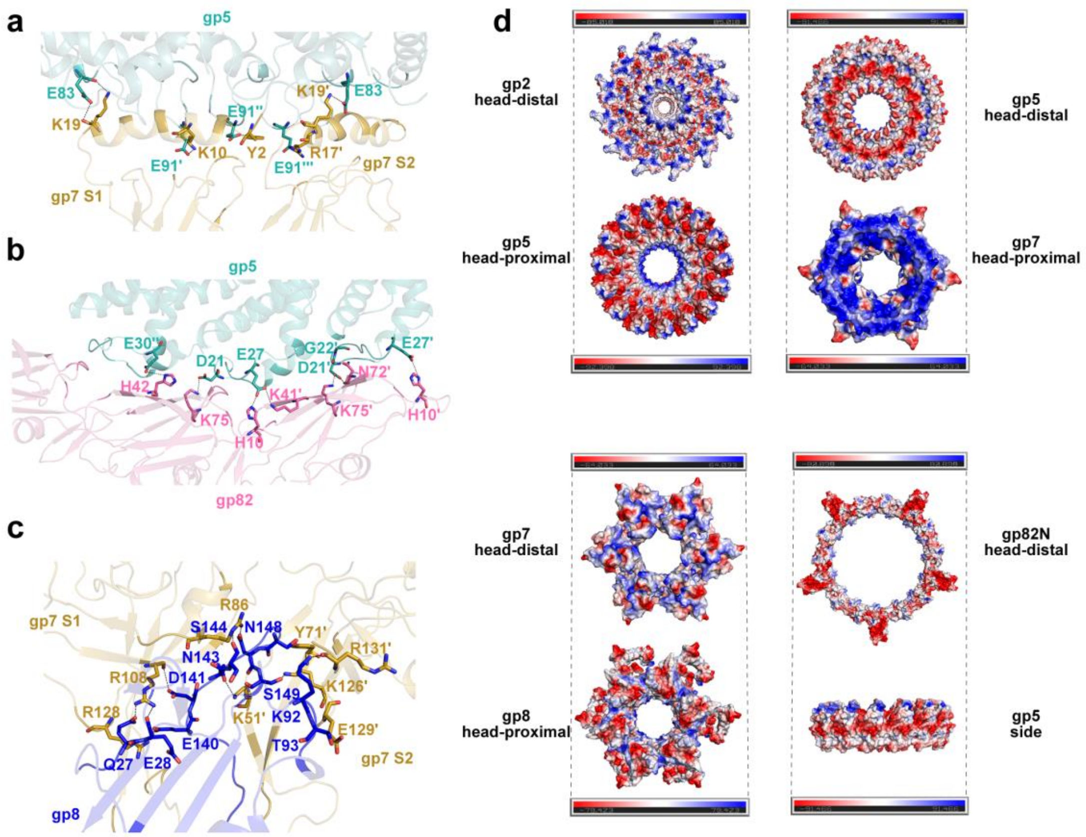

Supplementary Fig. 3 Interactions among the A-1(L) neck components and the terminator/gp8. a-c, Detailed interactions of gp5-gp7 (**a**), gp5-gp82N (**b**) and gp7-gp8 (**c**), corresponding to Fig. 2d-f. The interacting residues are shown as sticks and labeled, with hydrogen bonds indicated as dashed lines. **d,** Electrostatic potentials of the A-1(L) neck components and the terminator in head-proximal, head-distal or side view. All pairwise interfaces are generally complementary in shape and electrostatic potential, enabling the interlocked assembly of the symmetry-mismatched neck. Color scale bars of e-potential are shown in kT/e for each protein.

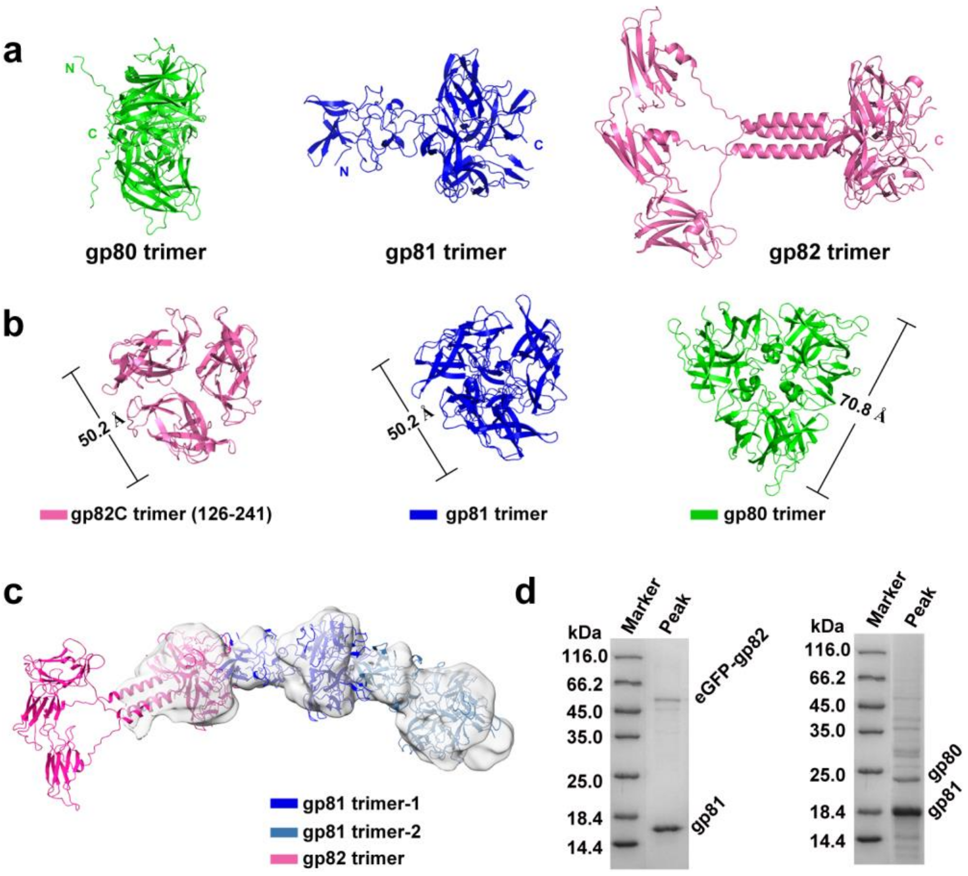

Supplementary Fig. 4 Structural prediction and analyses of the neck fiber proteins. **a,** AlphaFold2 predicted trimeric atomic models of proteins encoded by the neck fiber genes *gp80*, *gp81* and *gp82*. **b,** Diameters of trimeric gp82C, gp81 and gp80. gp82C: the C-terminal domain of gp82 (residues Ser126-Leu241). **c,** Predicted atomic models of one gp82 subunit and two gp81 subunits were fitted into a 6.07-Å cryo-EM map calculated from the recombinant neck fibers. **d,** SDS-PAGE profiles of the eGFP-gp82-gp81 (left) and gp81-gp80 (right) complexes from the highest peak fraction of corresponding gel filtration chromatography.

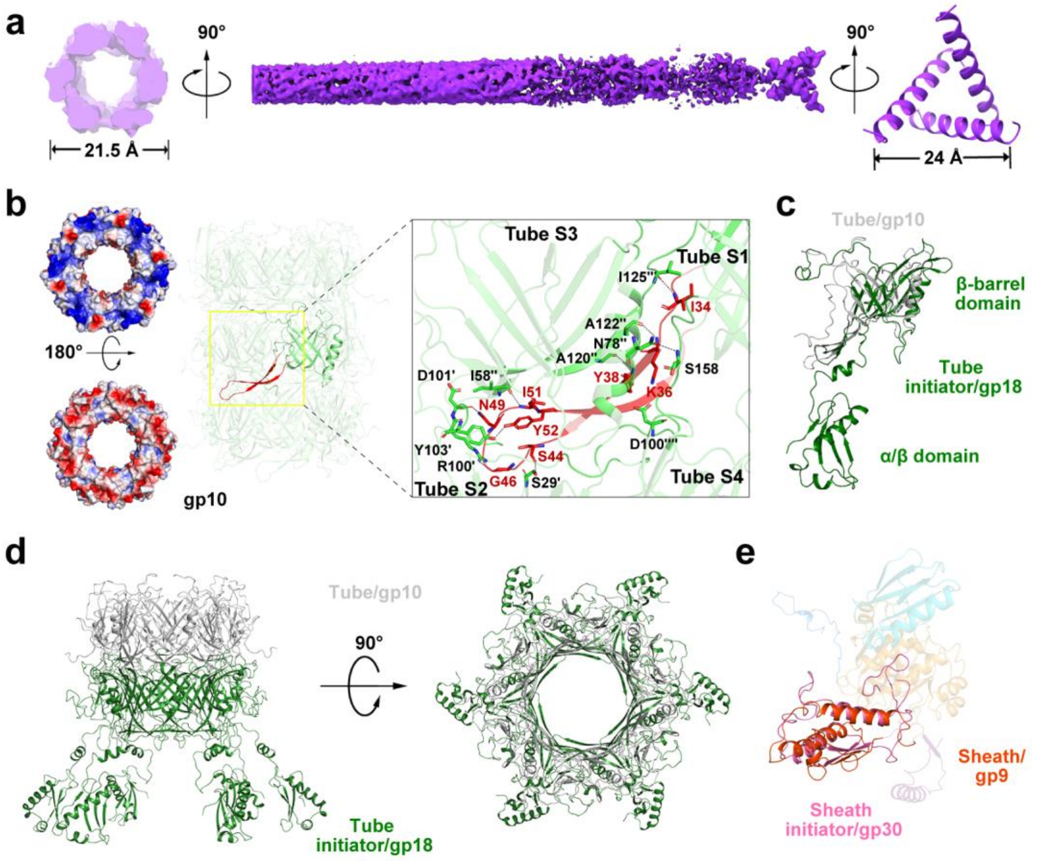

Supplementary Fig. 5 Structural analyses of the tube and sheath. a, The head-proximal, side and head-distal views of the TMP/gp13. The head-distal view shows the fitted three 20-residue C-terminal helices (residues Pro666-Ala689) of TMP that form a tripod structure. **b,** The helical organization of the tube. Three tube rings are displayed as semi-transparent cartoons, of which one tube subunit is highlighted as opaque cartoon with a red protruding β-hairpin. The inter-ring interaction is shown as an inset. The electrostatic potential of one tube ring is also shown in head-proximal and head-distal views, respectively. **c,** Superposition of the tube/gp10 (grey) against the tube initiator/gp18 (green). The two domains of gp18 are labeled. **d,** The first ring of the tube complexed with the tube initiator ring in side and head-proximal views. **e,** Superposition of the sheath/gp9 (with domains colored differently) against the sheath

initiator/gp30 (pink). Domain I, III and two folded termini of the sheath/gp9 are shown as semi-transparent cartoons, whereas gp30 and domain II of gp9 are shown as opaque cartoons.

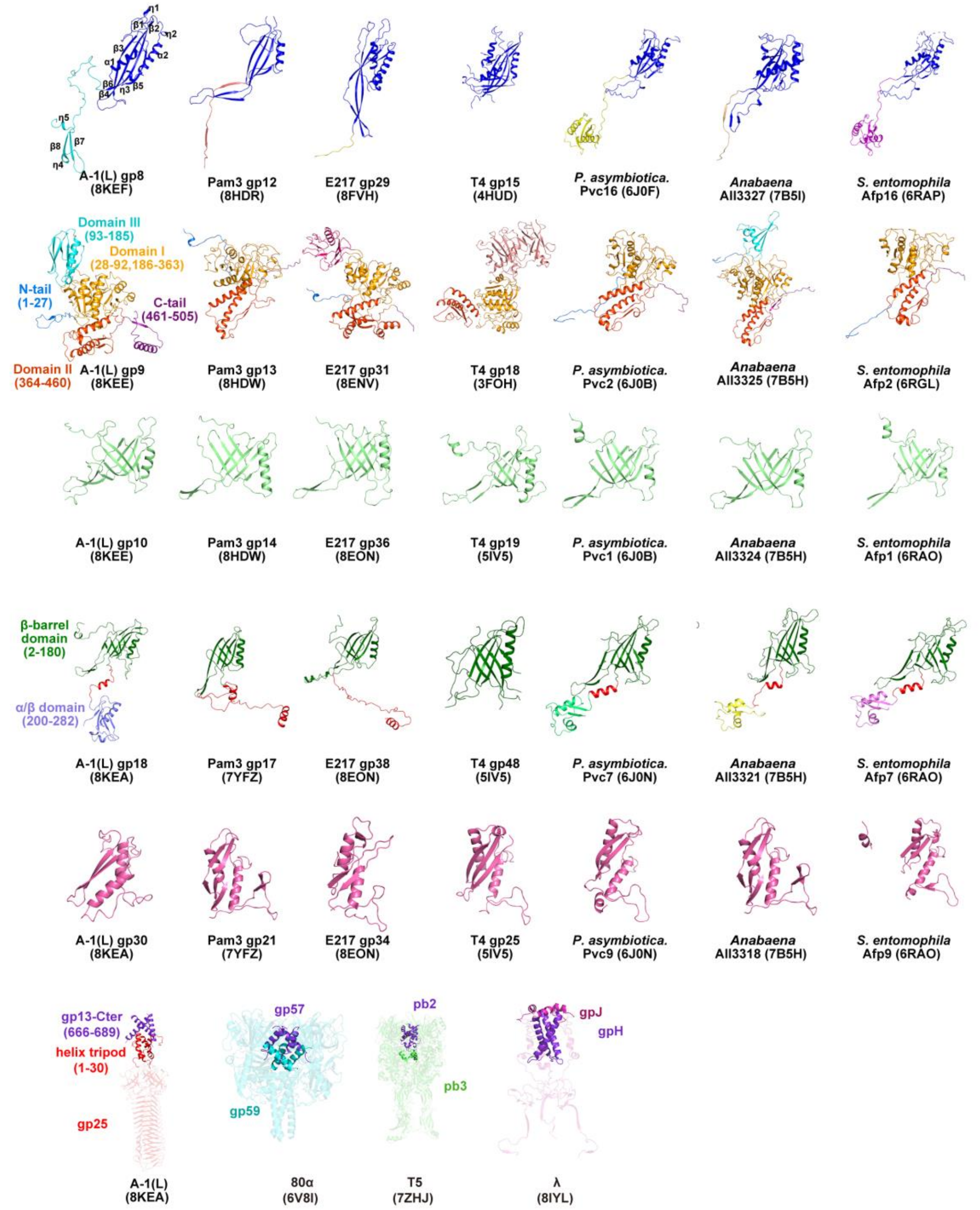

Supplementary Fig. 6 Structures of A-1(L) tail proteins and their homologs from different phages and bacterial extracellular contractile injection systems (eCISs). Three distal helices of TMP/gp13 usually form direct interactions with specific baseplate proteins, such as central spike and hub. The helices of baseplate proteins interacting with TMP are highlighted as opaque cartoons. The domains of the A-1(L)

tail proteins are labeled and colored differently. *P. asymbiotica*: *Photorhabdus asymbiotica*; *Anabaena*: *Anabaena* sp. PCC 7120; *S. entomophila*: *Serratia entomophila*.

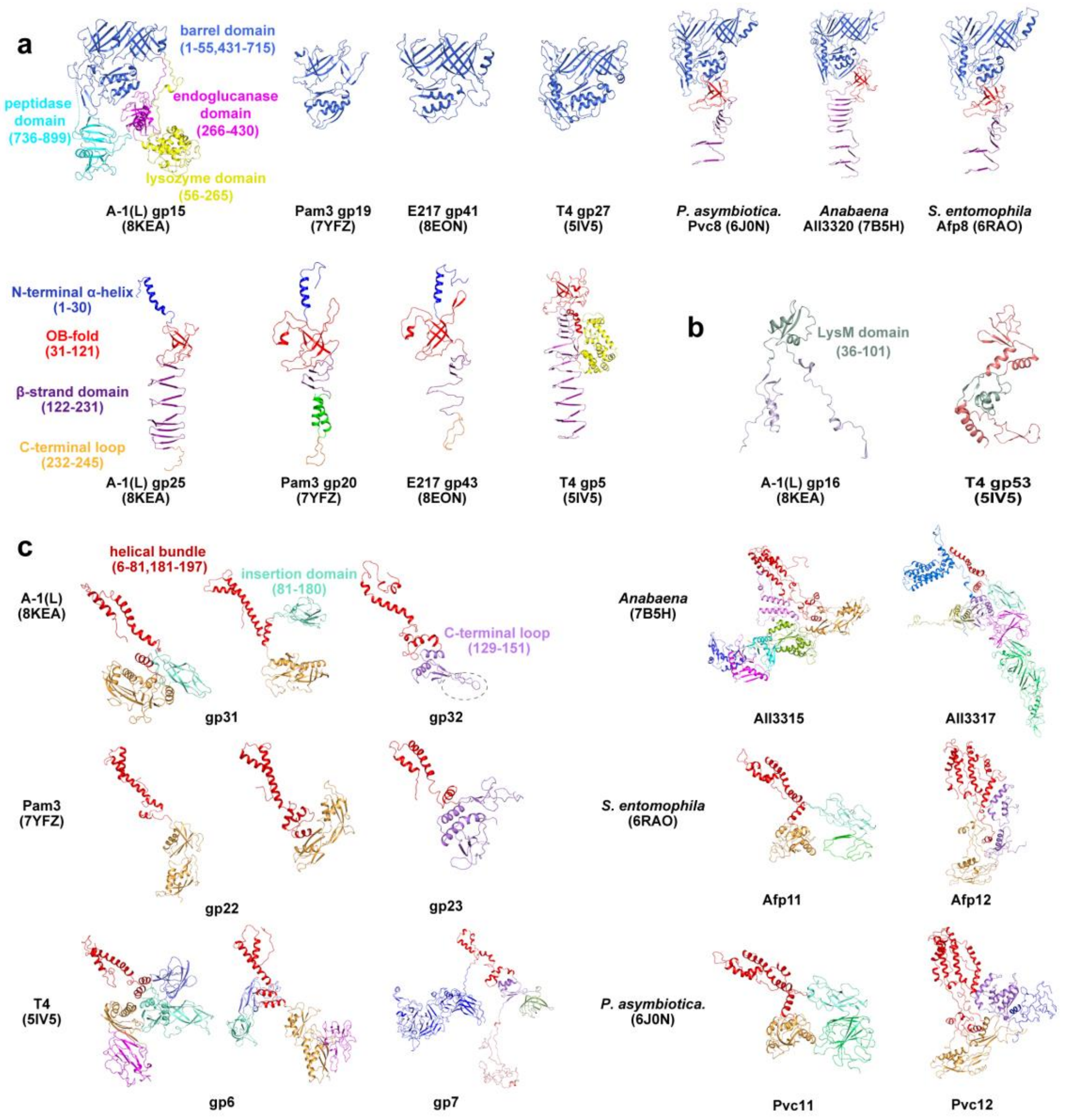

Supplementary Fig. 7 Structures of the baseplate proteins. a, Structures of A-1(L) hub/gp15 and central spike/gp25, in addition to their corresponding homologs from different phages and bacterial eCISs. Notably, the hub and central spike are normally expressed as one fusion protein in the bacterial eCISs. **b,** Structures of A-1(L) gp16 and another LysM-containing protein gp53 from the phage T4. **c,** Structures of the baseplate wedge from different phages and bacterial eCISs. The wedge subunits are shown as cartoons with domains colored differentially. The black circles indicate the C-terminal loop of gp32, which is involved in the interaction with the shoulder domain of LTF. The

domains of A-1(L) baseplate proteins are labeled and colored differently. *Anabaena*: *Anabaena* sp. PCC 7120; *S. entomophila*: *Serratia entomophila*; *P. asymbiotica*: *Photorhabdus asymbiotica*.

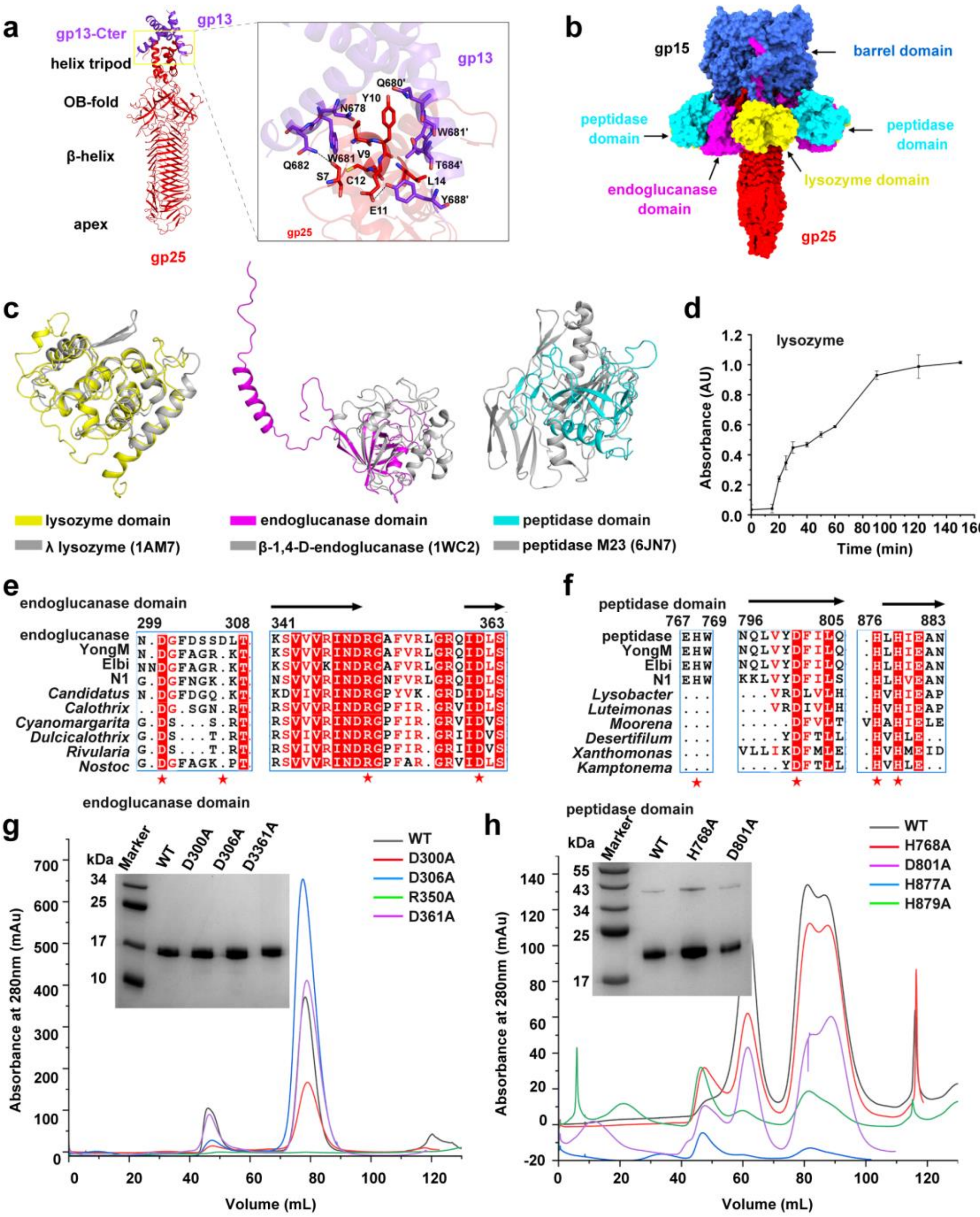

Supplementary Fig. 8 Structural analyses of the central spike and the hub. a, Cartoon presentations of trimeric central spike/gp25 in complex with the C-terminal tripod of TMP/gp13. The detailed interaction is shown as an inset. The domains of gp25 are labeled, of which OB-fold represents oligonucleotide/oligosaccharide binding fold. **b,** Side view of the trimeric hub in complex with the central spike. **c,** Superpositions of the lysozyme, endoglucanase and peptidase domains of the hub against λ lysozyme

(PDB:1AM7), β-1,4-D-endoglucanase (PDB: 1WC2) and peptidase M23 (PDB: 6JN7), respectively. **d,** Plot of cytochrome released from lytic *Anabaena* sp. PCC 7120 cells upon the treatment of commercially available lysozyme. The release of cytochrome was detected at the absorbance of 610 nm. Each data point is the average of three independent experiments (n = 3), and error bars represent the means ± SD. **e,f,** Multiple-sequence alignments of the hub endoglucanase (**e**) and peptidase (**f**) domain against various homologs. The residues involved in enzymatic catalysis are labeled with a red pentagram. **g-h**, Gel filtration chromatography and SDS-PAGE profiles of the endoglucanase (**g**) and peptidase (**h**) domain, in addition to their single mutants. Each sample for the SDS-PAGE analysis was collected from the highest peak fraction or fraction indicated by the arrow. WT: wild-type.

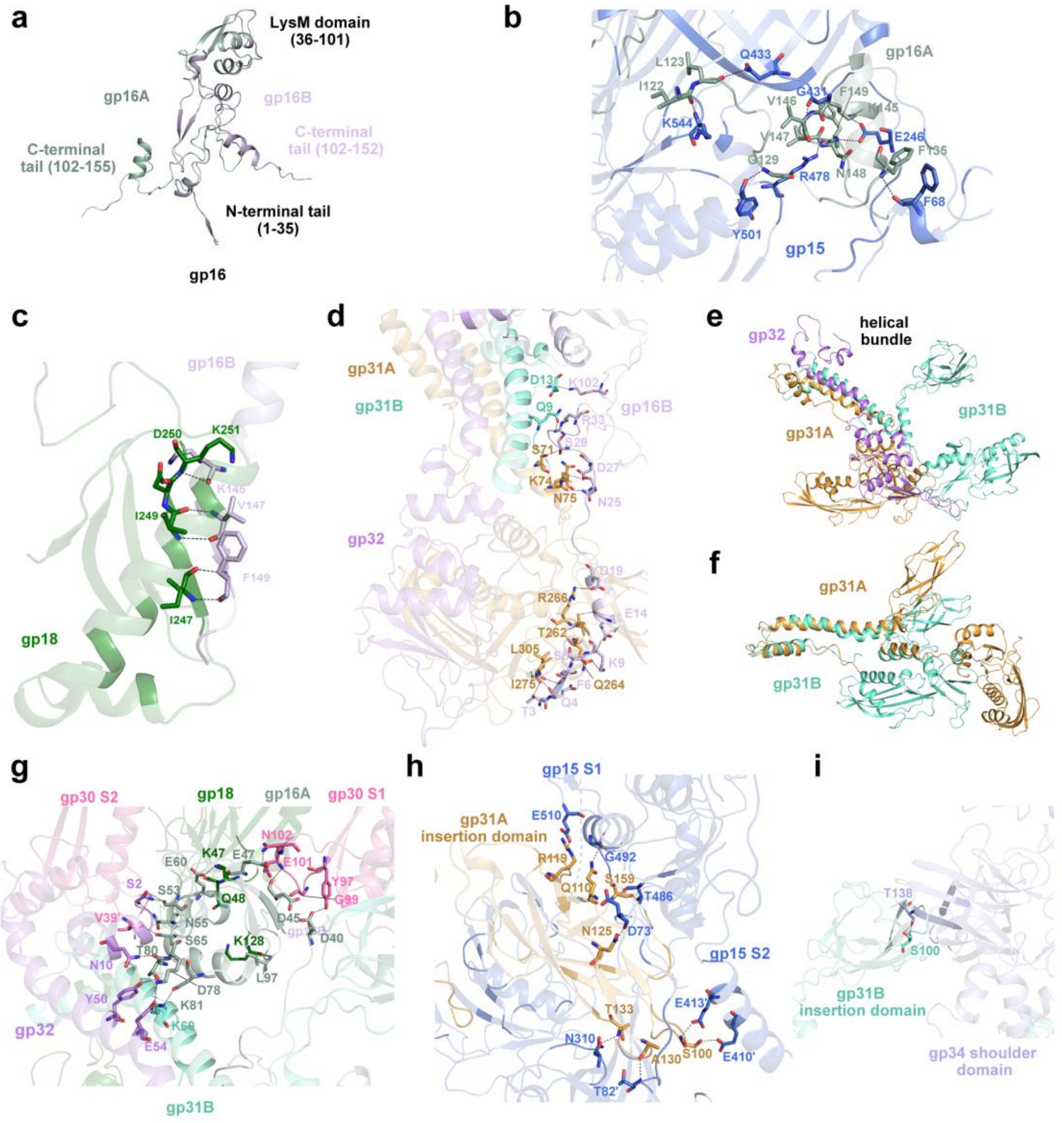

Supplementary Fig. 9 Structural analyses of the gp16 and wedge subunits, and the interactions among baseplate components. a, Superposition of the gp16A (green) against gp16B (pink). They share a same conformation of the N-terminal tail and LysM domain, but differ in the orientation at the C-terminal tail. **b-d,** The detailed interactions between gp16A C-terminal tail and the barrel domains of gp15 (**b**), gp16B C-terminal tail and the α/β domain of gp18 (**c**), in addition to that between gp16 N-terminal tail and heterotrimeric wedge subunits (**d**). **e,** Cartoon presentation of the heterotrimeric wedge. gp31A, gp31B and gp32 are colored in sandy brown, cyan and purple,

respectively. The N-terminal three helices of gp31 and gp32 form a helical bundle. **f,** Superposition of gp31A (sandy brown) and gp31B (cyan). The N-terminal helices could be well aligned. **g**, The detailed interactions of the LysM domain of gp16A with the N-terminal helical bundle of baseplate wedge, two gp30 subunits and one gp18 subunit. **h,i,** The detailed interactions between the insertion domain of gp31A and the hub (**h**), and between the insertion domain of gp31B and the LTF/gp34 (**i**). The detailed interactions in panels **b-d** and **g-i** are corresponding to those in Fig. 5f-h.

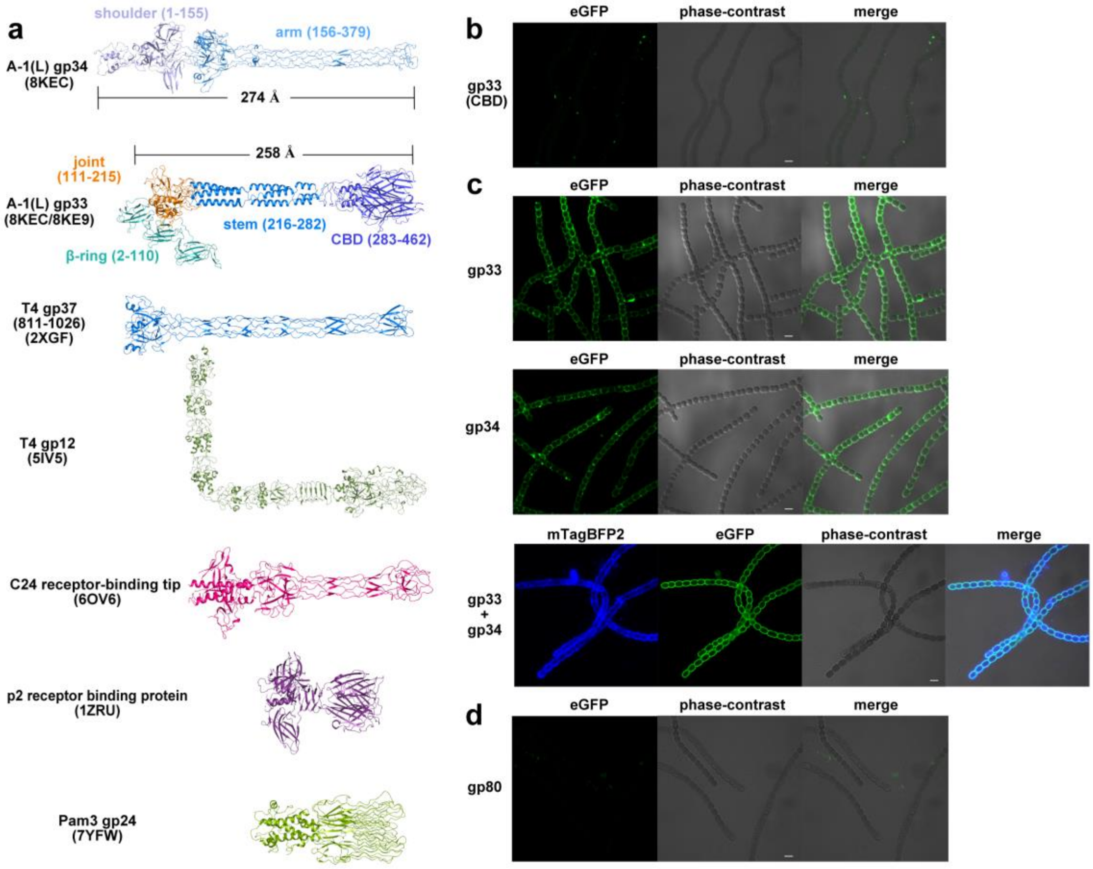

Supplementary Fig. 10 Structural and functional analyses of the tail fibers. a, Structures of the trimeric tail fibers from different phages. The domains and the lengths of A-1(L) gp34 and gp33 are labeled and colored differently. **b,** Confocal image of gp33 (CBD) incubating with *Anabaena* sp. PCC 7120 cells. **c,** Confocal images of recombinant full-length gp33 and gp34 binding to *Anabaena* sp. PCC 7120 cells. The green fluorescence (eGFP) is excited by the wavelength of 488 nm, whereas that for the blue fluorescence (mTagBFP2) is 405 nm. For the competition binding assays, gp33 and gp34 are fused with eGFP and mTagBFP2, respectively. Magnification, 100×. Scale bar: 5 μm. **d,** Confocal image of neck fiber/gp80 incubating with *Anabaena* sp. PCC 7120 cells.

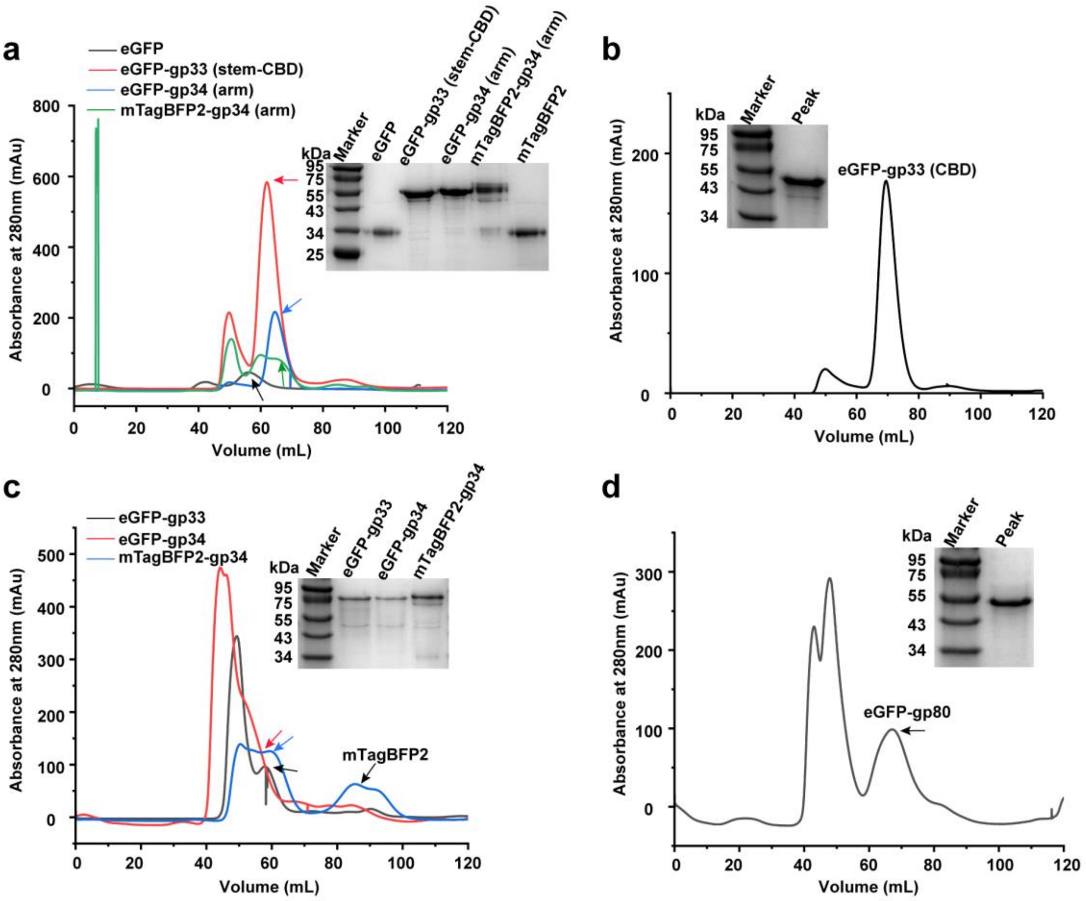

Supplementary Fig. 11 Gel filtration chromatography and SDS-PAGE profiles of proteins used in the binding assays. a, Profiles of purified eGFP and different gp33 and gp34 constructs. **b,** Profile of purified eGFP-gp33 (CBD). **c,** Profiles of different tagged full-length gp33 and gp34. **d,** Profile of purified eGFP-gp80. Each sample for the SDS-PAGE analysis was collected from the highest peak fraction or fraction indicated by the arrow.

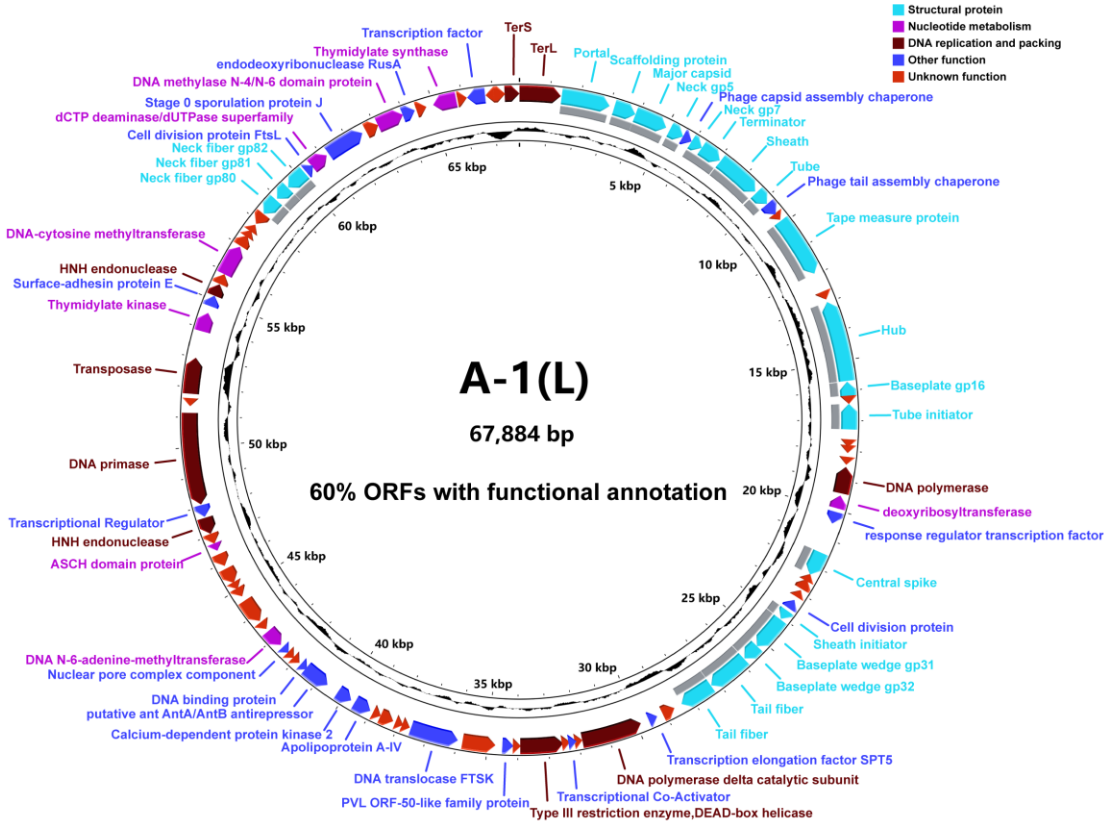

Supplementary Fig. 12 The revised circular genomic map of A-1(L). Circles from the outmost to the innermost correspond to the following: (i) predicted ORFs with known functions are labeled and colored based on their functions; (ii) structural proteins identified by mass spectrometry are shown by gray lines, and (iii) G + C content plotted relative to the genomic mean of 36.51% G + C.

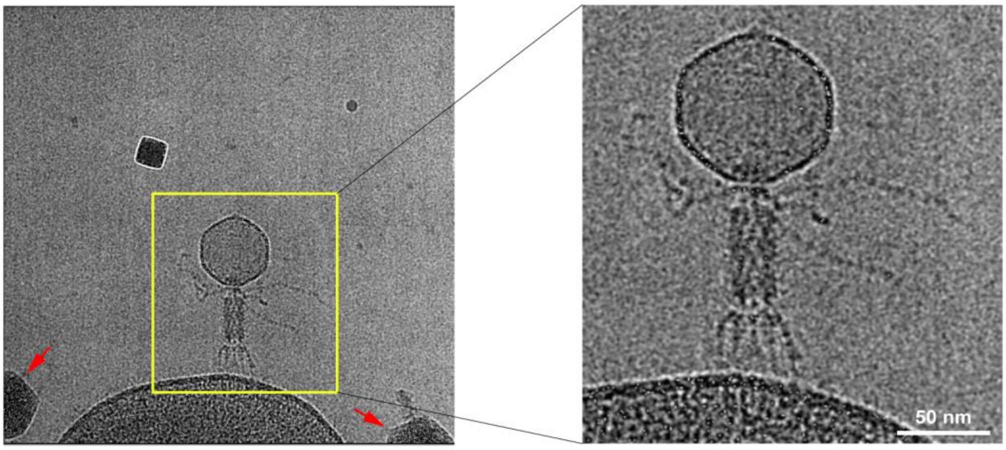

Supplementary Fig. 13 A representative cryo-EM image of the A-1(L) particle. The image shows the A-1(L) particle with the tail fibers binding to the co-purified versicles. At this state, the tail fibers have been released from the sheath. Red arrows indicate the capsid of mature A-1(L) particles. Scale bar: 50 nm.

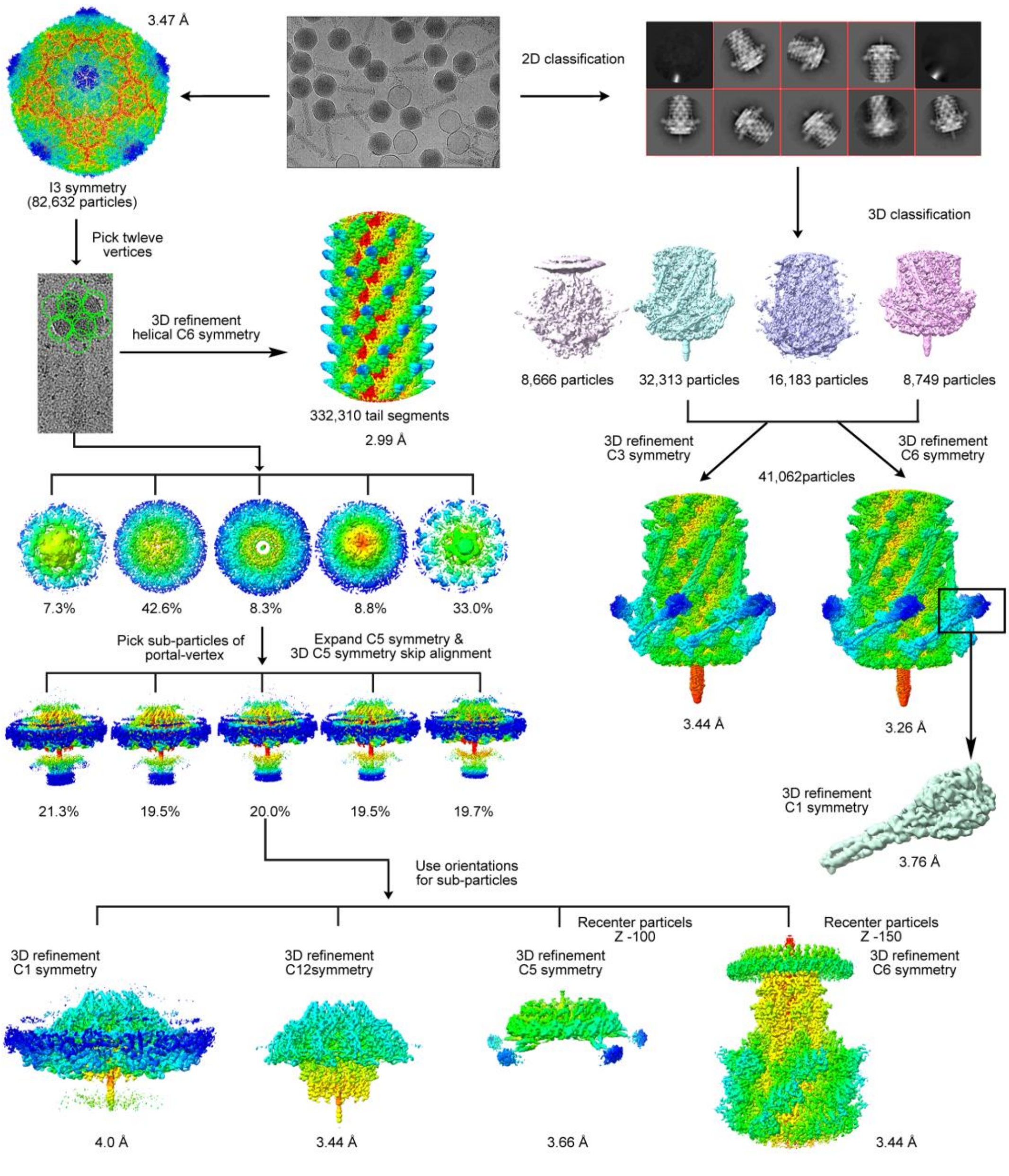

Supplementary Fig. 14 The flowchart of cryo-EM data processing of the A-1(L) virion. The sequential localized classification and subparticle reconstruction of the A-1(L) capsid, neck, tail and baseplate are presented as a flowchart.

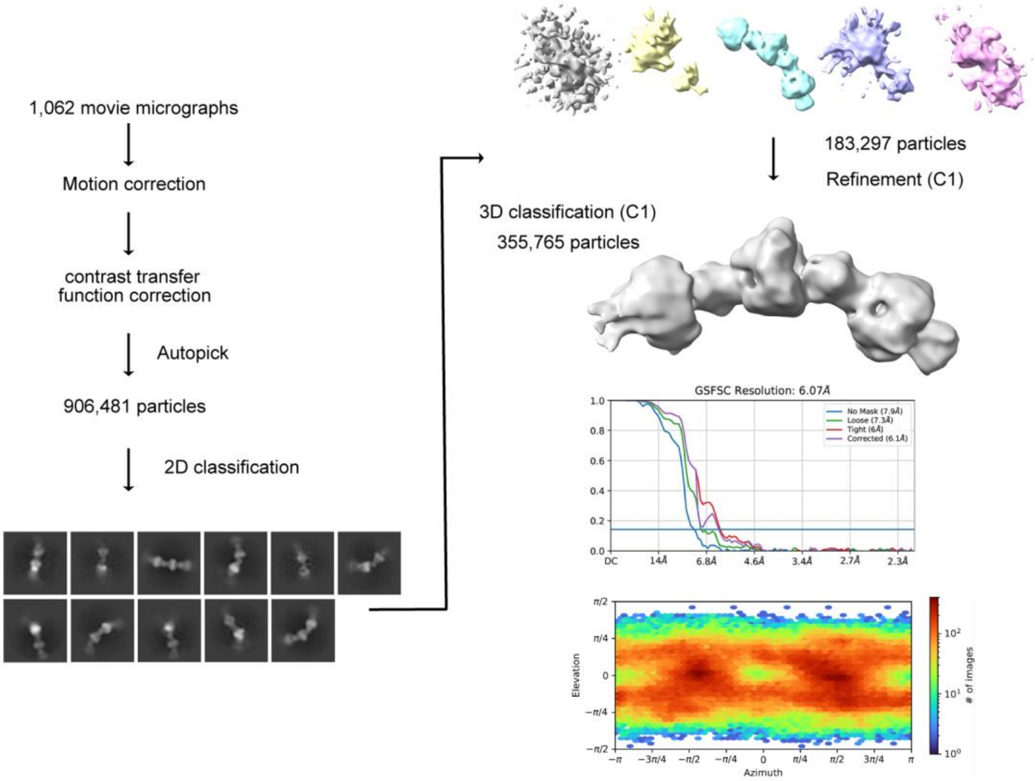

Supplementary Fig. 15 The flowchart of cryo-EM data processing of the recombinant neck fibers. Fourier shell correlation (FSC) curve is also displayed.

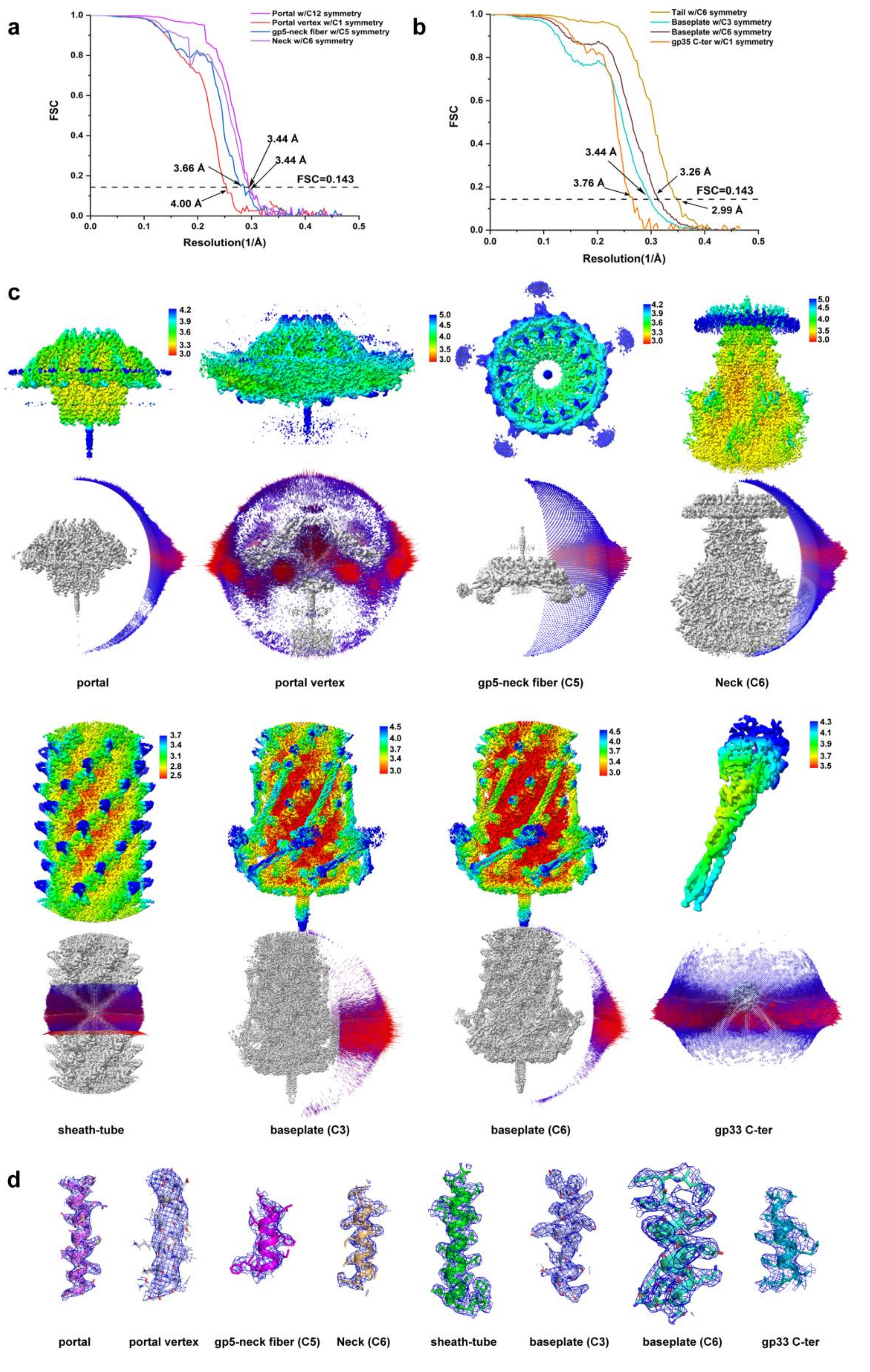

Supplementary Fig. 16 Resolutions of the A-1(L) virion. a,b, The gold-standard FSC

curves of the corresponding cryo-EM maps of neck (**a**) in addition to tail and baseplate (**b**). **c,** The local resolution maps of different parts of the A-1(L) virion, and the corresponding euler angle distribution of the classified particles used for the final 3D refinement of the overall map. **d,** Cryo-EM maps for representative segments of the different maps.

Supplementary Table 1: Predicted ORFs of cyanophage A-1(L).

ORF	Direction	Start	Stop	Length (bp)	Length (AA)	MS	Function protein	Related phages or microbes	E-Value[1]	Probability[2]
1	F	1	1359	1359	452		TerL[1]	*Nostoc* phage YongM	0	
2	F	1389	2993	1605	534	+	Portal[3]			
3	F	3147	3878	732	243	+	Scaffolding protein[2]	*Bacillus* phage SPP1		97.14
4	F	3884	4981	1098	365	+	Major capsid[3]			
5	F	5120	5608	489	162	+	Neck gp5[3]			
6	F	5605	5931	327	108		Putative capsid assembly protein[2]	*Escherichia* phage Mu		98.67 (4.1e-7)
7	F	5931	6329	399	132	+	Neck gp7[3]			
8	F	6326	7048	723	240	+	Terminator[3]			
9	F	7080	8600	1521	506	+	Sheath[3]			
10	F	8657	9160	504	167	+	Tube[3]			
11	F	9172	9612	441	146		Phage tail assembly chaperone[2]	Bacteriophage HK97		86.02(5.3)
12	F	9602	9814	213	70					
13	F	9884	11953	2070	689	+	Tape measure protein[3]			
14	F	12606	12893	288	95					
15	R	12979	15678	2700	899	+	Hub[3]			
16	R	15703	16169	468	155	+	Baseplate gp16[3]			
17	F	16168	16449	282	93					
18	R	16439	17287	849	282	+	Tube initiator[3]			
19	F	17654	17851	198	65					
20	F	17854	18033	180	59					
21	F	18263	18403	141	46					

22	R	18536	19426	891	296		Exonuclease RNase T and DNA polymerase/DNA polymerase III, alpha subunit[1]	*Candidatus Aminicenantes*	8.00E-15	
23	R	19500	19937	438	145		Deoxyribosyltransferase[2]	*Lactobacillus helveticus* H10		99.72
24	F	20064	20468	405	134		response regulator transcription factor[1]	*Streptosporangiaceae*	1.00E-04	
25	F	21498	22310	813	270	+	Central spike[3]			
26	R	22366	22473	108	35					
27	R	22485	22859	375	124					
28	R	22926	23144	219	72					
29	R	23292	23621	330	109		Cell division protein[2]	*Escherichia coli*		95.33
30	F	23717	24061	345	114	+	Sheath initiator[3]			
31	F	24084	25256	1173	390	+	Baseplate wedge gp31[3]			
32	F	25256	25831	576	191	+	Baseplate wedge gp32[3]			
33	F	25853	27241	1389	462	+	Tail fiber[3]			
34	F	27257	28396	1140	379	+	Tail fiber[3]			
35	R	28666	29094	429	142					
36	R	29288	29596	309	102		Transcription elongation factor SPT5[2]	*Homo sapiens*		92.83
37	R	29870	31876	2007	668		DNA polymerase delta catalytic subunit[1]	cyanobacterium TDX16	2.00E-134	
38	R	31903	32094	192	63					
39	R	32097	32309	213	70		Transcriptional Co-Activator[2]	*Homo sapiens*		88.1
40	R	32309	32518	210	69					
41	R	32502	33917	1416	471		Type III restriction enzyme,DEAD-box helicase[1]	*Iphinoe* sp. HA4291-MV1	7.00E-129	
42	R	33914	34144	231	76					

43	R	34141	34506	366	121	PVL ORF-50-like family protein[2]			98.44(0.0000018)
44	R	34735	35823	1089	362				
45	R	35974	37566	1593	530	DNA translocase FTSK[1]	*Nostoc* phage YongM	0	
46	R	37606	37881	276	91				
47	R	37881	38075	195	64				
48	R	38191	38643	453	150				
49	R	38633	38917	285	94				
50	R	39013	39597	585	194	Apolipoprotein A-IV[2]	*Homo sapiens*		94.15(3.7)
51	R	39732	40247	516	171	Calcium-dependent protein kinase 2[2]	*Plasmodium falciparum* K1		85.66(39)
52	R	40666	41556	891	296	putative ant AntA/AntB antirepressor[1]	*Nostoc piscinale*	3.00E-32	
53	R	41570	41713	144	47	DNA binding protein[2]	*Myxococcus xanthus* DK 1622		99.18 (1.5e-9)
54	R	41856	42092	237	78				
55	R	42089	42343	255	84				
56	R	42340	42582	243	80	Nuclear pore complex component[2]			87.38(6.6)
57	R	42689	43306	618	205	DNA N-6-adenine-methyltransferase[1]	*Pseudoalteromonas* sp. 2CM39R	9.00E-09	
58	R	43400	43690	291	96				
59	R	43739	44575	837	278				
60	R	44738	45061	324	107				
61	R	45048	45266	219	72				
62	R	45263	45799	537	178				
63	R	45899	46333	435	144				
64	R	46465	46707	243	80	ASCH domain protein[1]	*Coprococcus comes*	3E-17	
65	R	46709	47059	351	116				
66	R	47056	47619	564	187	HNH endonuclease[1]	*Bacillus* sp. NTK034	1.00E-12	

67	R	47653	48057	405	134		DNA-binding domain(AP2)/Transcriptional Regulator[2]	*Plasmodium falciparum* 3D7		98.04(0.00005)
68	R	48079	51144	3066	1021		virulence-associated E family protein[1]/DNA primase[2]	*Scytonema* sp. UIC 10036[1]/ *Staphylococcus aureus*[2]	4.00E-28	100(7.7E-30)
69	R	51378	51620	243	80					
70	F	51778	52986	1209	402		Transposase[1]	*Nostoc* sp. PCC 7120 = FACHB-418	0	
71	F	53883	54521	639	212		Thymidylate kinase[1]	*Dactylococcopsis salina* PCC 8305	1.00E-32	
72	F	54758	55132	375	124		Surface-adhesin protein E[2]			93.7(0.51)
73	F	55129	55545	417	138		HNH endonuclease[1]	*Moorena* sp. SIO3I7	9.00E-08	
74	F	55555	55914	360	119					
75	F	55911	56954	1044	347		DNA-cytosine methyltransferase[1]	*Janthinobacterium svalbardensis*	5.00E-76	
76	F	56986	57399	414	137					
77	F	57399	57566	168	55					
78	F	57598	57813	216	71					
79	R	57877	58287	411	136					
80	R	58301	58906	606	201	+	Neck fiber gp80[4]			
81	R	58959	59465	507	168	+	Neck fiber gp81[2/4]	*Bacillus* phage phi29		97.96(0.00087)
82	R	59478	60203	726	241	+	Neck fiber gp82[2/3/4]	*Bacillus* phage phi29		97.58(0.0047)
83	F	60265	60567	303	100		Cell division protein FtsL[2]			89.39(0.39)
84	F	60530	61129	600	199		dCTP deaminase/dUTPase superfamily[1]	*Nostoc* phage YongM	2.00E-95	
85	F	61216	62514	1299	432		Stage 0 sporulation protein J/ParB family protein[2]	*Myxococcus xanthus* DK 1622		99.9(7.7E-22)
86	F	62653	63099	447	148					
87	F	63089	63976	888	295		DNA methylase N-4/N-6 domain protein[1]	*Seinonella peptonophila*	7.00E-93	

88	F	63966	64397	432	143	endodeoxyribonuclease RusA[1]	*Iphinoe* sp. HA4291-MV1	4.00E-09	
89	F	64447	64791	345	114				
90	R	65016	65795	780	259	Thymidylate synthase[1]	*Vicingus serpentipes*	2.00E-53	
91	F	65839	66153	315	104				
92	R	66156	66752	597	198	Transcription factor[2]	*Pyrococcus furiosus*		95.8(0.067)
93	R	66802	67002	201	66				
94	F	67036	67419	384	127				
95	F	67397	1	489	162	TerS[2]	*Shigella* phage Sf6		98.41(0.0000072)

[1] predicted by BLASTp searching against the NCBI nr database.

[2] predicted by HHpred tool.

[3] solved by cryo-electron microscopy.

[4] predicted by AlphaFold 2.0.

+ identified by mass spectrometry (MS).

Supplementary Table 2: Sequence comparison of present genome against previously reported genome.

Present region	Present nucleotide sequences	Coding sequence	Previous nucleotide sequences	Previous region
14,421-14,436 (*gp15*)	CTGGTTTTTTTTAATC	Y	CTGGTTTTTTTAATC	14,421-14,435 (*gp16*)
16,600-16,616 (*gp18*)	TATATTTTTTTTCAAAT	Y	TATATTTTTTTCAAAT	16,599-16,614 (*gp19*)
20,942-20,953 (*gp24-gp25*)	GTAACGCGAAAA	N	GTAACGTGGAAA	20,940-20,951 (*gp25-gp26*)
24,801-24,815 (*gp31*)	CATCTTTTTTTATTG	Y	CATCTTTTTTATTG	24,798-24,811 (*gp32*)
27,842-27,856 (*gp34*)	CTTTATTTGTTTTAT	Y	CTTTATTTGCTTTAT	27,839-27,853 (*gp36*)
28,630-28,644 (*gp34-gp35*)	CACAGTGGATAGATA	N	CACAGTGGANNTAGATA	28,627-28,643 (*gp36-gp37*)
46,400-46,414 (*gp63-gp64*)	CTAAAAAAAAAAACTC	N	CTAAAAAAAAAACTC	46,399-46,413 (*gp65-gp66*)
53,912-53,913 (*gp70-gp71+gp71*)	TATGCAGGTACTTTC	N+Y	TATGCAGGTACTTTCATCGTTTTTACGTCGTTCAAAATTCATAGAAACGAAAATTCATTTTTGTACTTAGTGTACTGATACGTATACTTATATACTATGTATATTTATACTATTAAGTATTTAAGTATATATATTTCATCTATATATTTAACTATAAAAACGTAACATTGTAACAAAAACGGCTAGAACCCTTTCCCTATCTAGATTTCAGCGAATTACAAATCCGTTACAACCTGTTACGTTTTTGAAATTTGTAACGTTACGGTTTTTGGGCTAAACGCCCCGGAGTCAGTCACAGCAACGGTTCCACGCTGTTACATTTTTGCGTTACAAATTTCTGCCAAAATGTAACGCCGATTTG	53,911-54,334 (*gp72-gp73+gp73*)

			TAACGCTGTGATATTCTGATAAAAACTTTGGA<br>GAACGATATGAACACCAGACAAGAAAAACCC<br>TATGCAGGTACTTTC	
58,904-58,918<br>(*gp80-gp81*)	ATATTTTTTTTTATT	N	ATATTTTTTTTATT	59,326-59,339<br>(*gp82-gp83*)
61,189-61,203<br>(*gp84-gp85*)	TATTTTTTTTTTAGG	N	TATTTTTTTTTAGG	61,610-61,623<br>(*gp86-gp87*)

Supplementary Table 3: Cryo-EM parameters, data collection and refinement statistics.

Data collection and processing	A-1(L) portal (PDB 8TS6) (EMD-41590)	gp5-neck fiber (PDB 8KEG) (EMD-37155)	Neck (PDB 8KEF) (EMD-37154)	Sheath-tube (PDB 8KEE) (EMD-37153)	Baseplate (PDB 8KEA) (EMD-37151)	Tail fiber (PDB 8KEC) (EMD-37152)	gp33 CBD (PDB 8KE9) (EMD-37150)
Magnification	81,000	81,000	81,000	81,000	81,000	81,000	81,000
Voltage (keV)	300	300	300	300	300	300	300
Electron exposure (e^-/Å^2)	50	50	50	50	50	50	50
Defocus range (μm)	-1.5~-2.5	-1.5~-2.5	-1.5~-2.5	-1.5~-2.5	-1.5~-2.5	-1.5~-2.5	-1.5~-2.5
Pixel size (Å)	1.07	1.07	1.07	1.07	1.07	1.07	1.07
Symmetry imposed	C12	C5	C6	C6	C3	C6	C1
Initial particle images (no.)	85,553	85,553	85,553	73,159	73,159	73,159	246,372
Final particle images (no.)	66,281	36,158	63,036	41,062	41,062	41,062	246,372
Map resolution (Å)	3.44	3.66	3.44	3.26	3.44	3.90	3.76
FSC threshold	0.143	0.143	0.143	0.143	0.143	0.143	0.143
Map resolution range (Å)	2.14~999	2.14~999	2.14~999	2.14~999	2.14~999	2.14~999	2.14~999
**Refinement**							
Real-space correlation coefficient	0.86	0.81	0.74	0.86	0.86	0.84	0.78
Initial model used (PDB code)	*ab-initio*	*ab-initio*	*ab-initio*	*ab-initio*	*ab-initio*	*ab-initio*	*ab-initio*
Map sharpening B factor (Å^2)	-136.654	-127.262	-116.259	-60.391	-62.564	-62.564	-15
**Model composition**							
Nonhydrogen atoms	46,752	30,930	17,802	91,279	97,165	87,756	4,929
Protein residues	5,772	3,855	2,226	12,011	12,456	11,838	654
Waters	0	0			0		
**RMS deviation from ideality**							
Bond lengths (Å)	0.004	0.003	0.016	0.004	0.005	0.004	0.004
Bond angles (°)	0.875	0.887	2.016	1.075	1.065	0.618	0.998
**Validation**							
MolProbity score	2.20	2.87	1.96	1.94	2.32	2.30	2.68
Clash score	9.46	21.34	8.10	5.84	11.31	11.08	12.52
Poor rotamers (%)	4.83	4.91	1.44	2.89	5.28	3.29	7.55
**Ramachandran statistics**							

Favored regions (%)	96.97	91.70	94.01	96.05	96.76	94.95	94.14
Allowed regions (%)	2.82	8.80	5.99	3.68	3.16	4.85	5.71
Outliers (%)	0.21	0.13	0.00	0.28	0.08	0.20	0.15
