# Supplementary material for: Structure of the intact tail machine of Anabaena myophage A-1(L)
Source: Nat Commun. 2024 Mar 26;15:2654. doi: 10.1038/s41467-024-47006-z (PMC10966104; doi:10.1038/s41467-024-47006-z)
Supplement: Supplementary file 4 — Source Data [file 41467_2024_47006_MOESM4_ESM.zip › Uncropped Gels.docx]

**Source Data files**

Uncropped gel for Fig. 3b


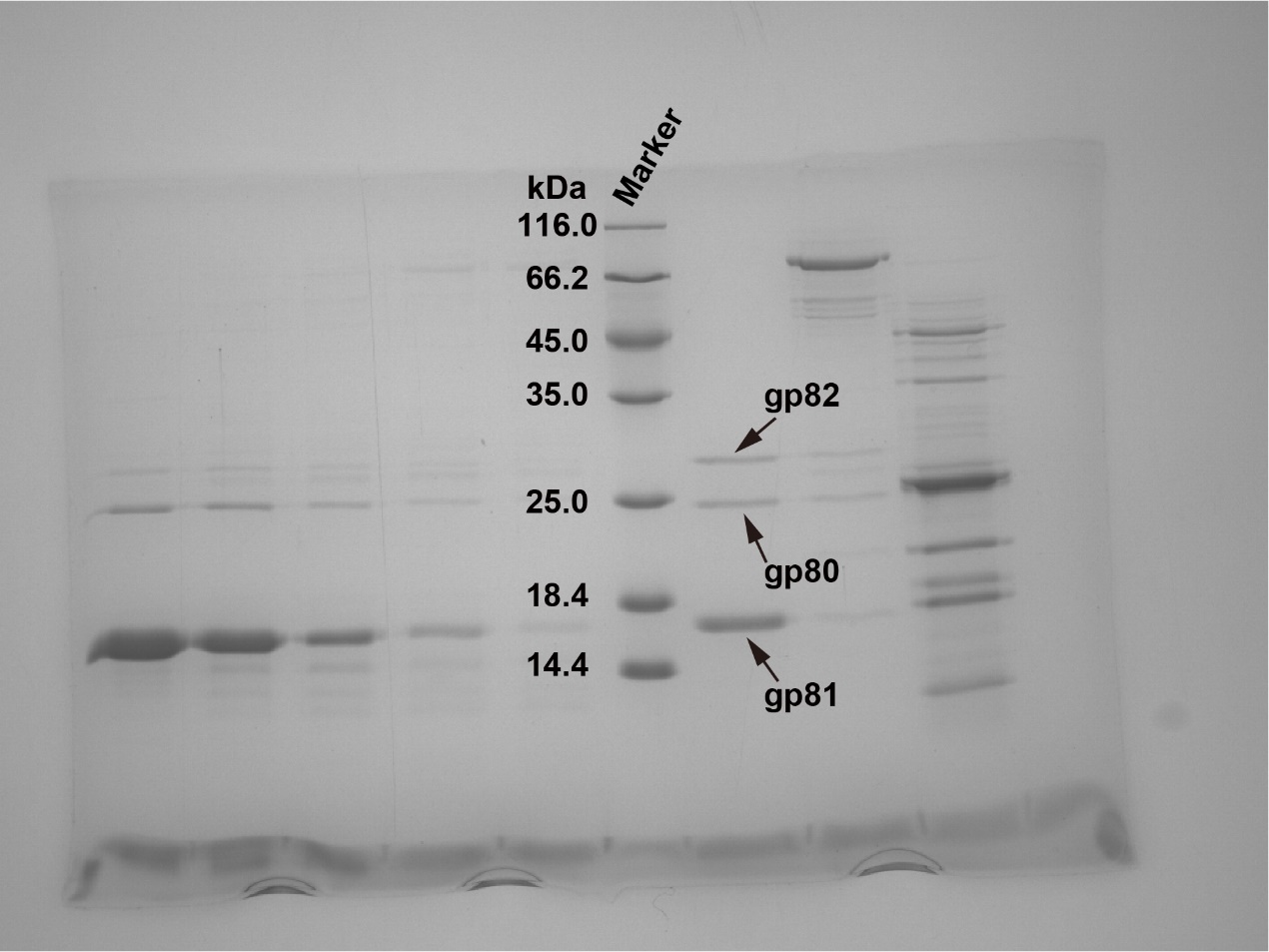


Uncropped gel for Supplementary Fig. 4d


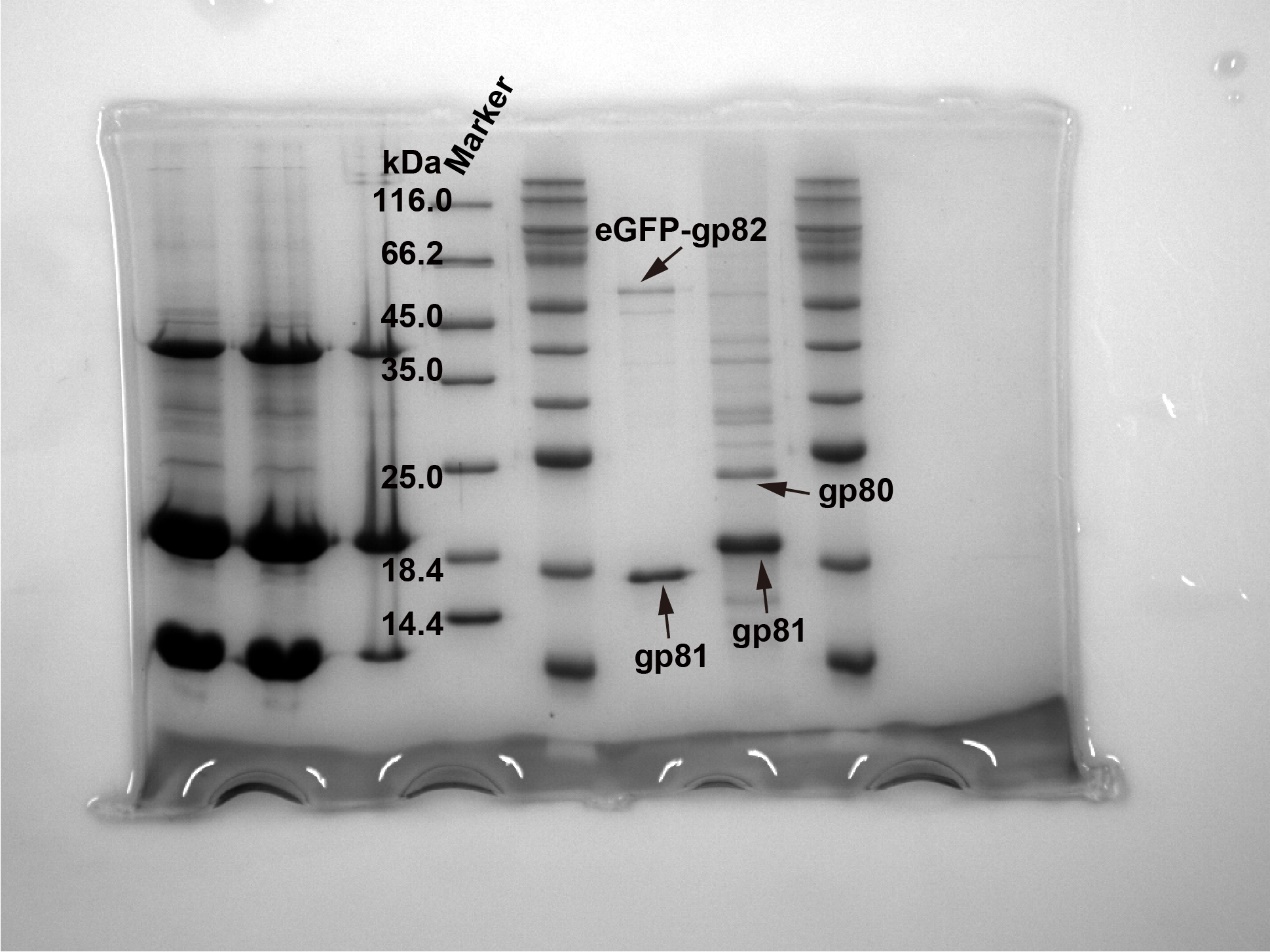


Uncropped gel for Supplementary Fig. 8g-h


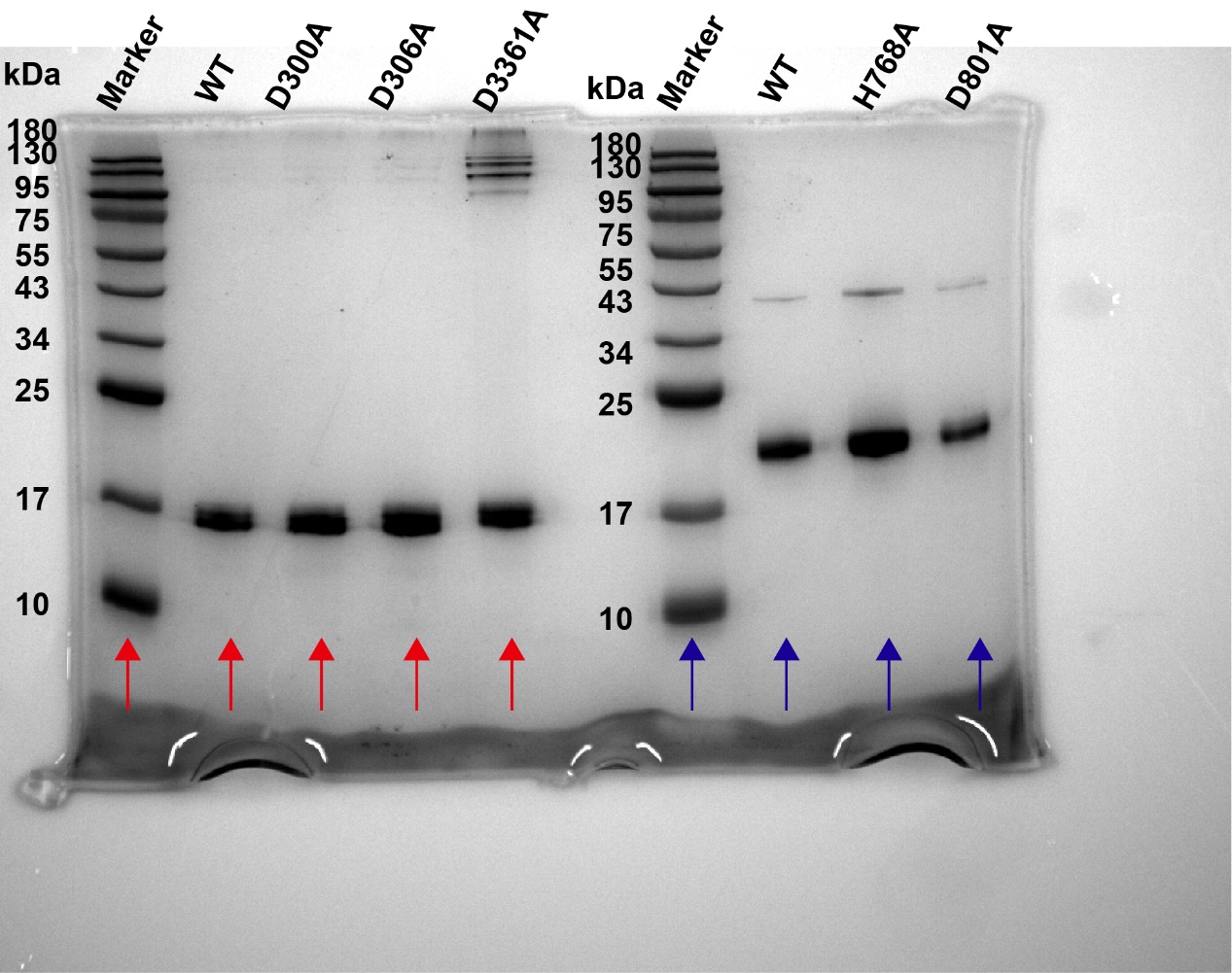


The cropped lanes were indicated by red (S8g) and blue (S8h) arrows, respectively.

Uncropped gel for Supplementary Fig. 11a


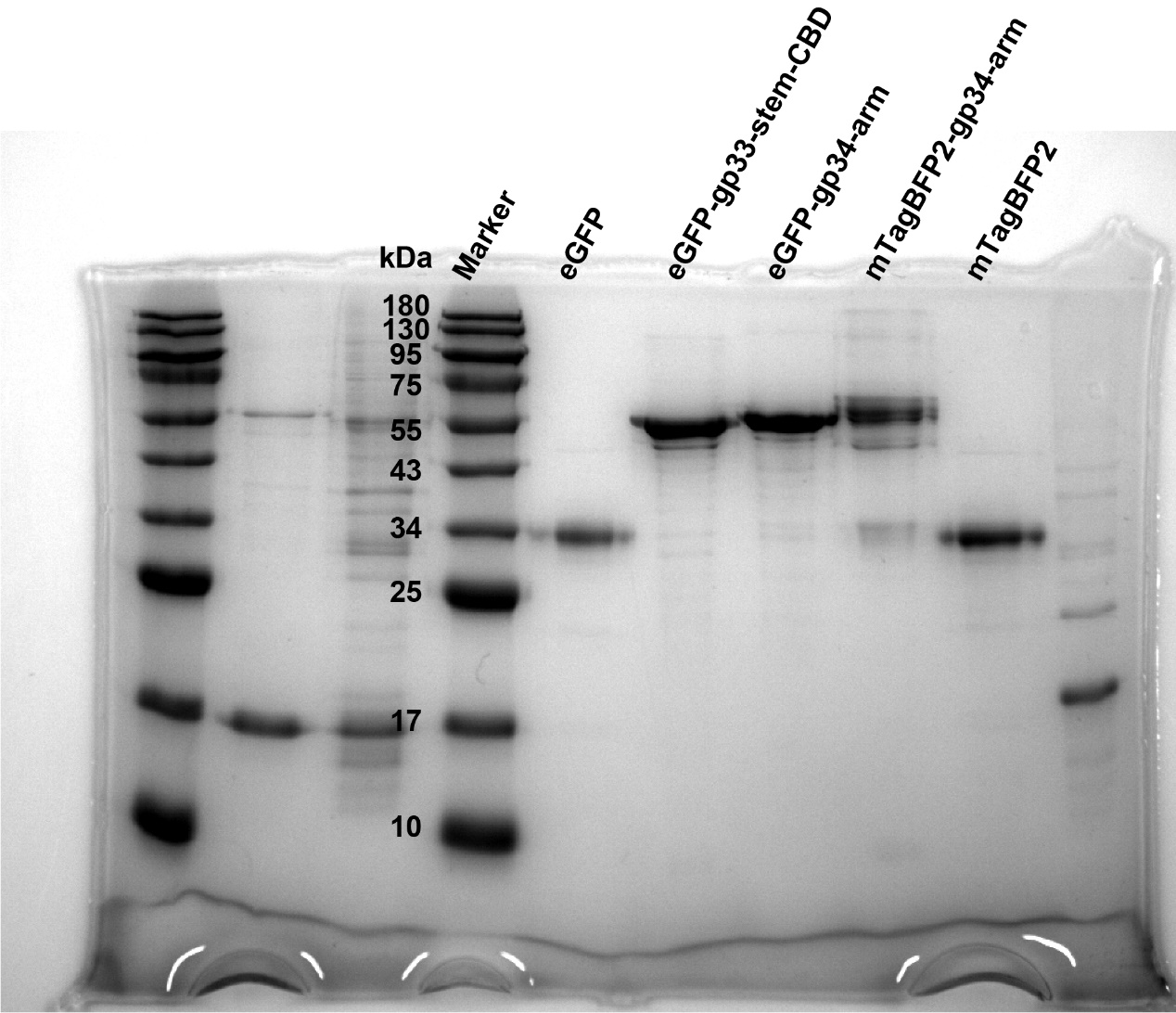


Uncropped gel for Supplementary Fig. 11b-d


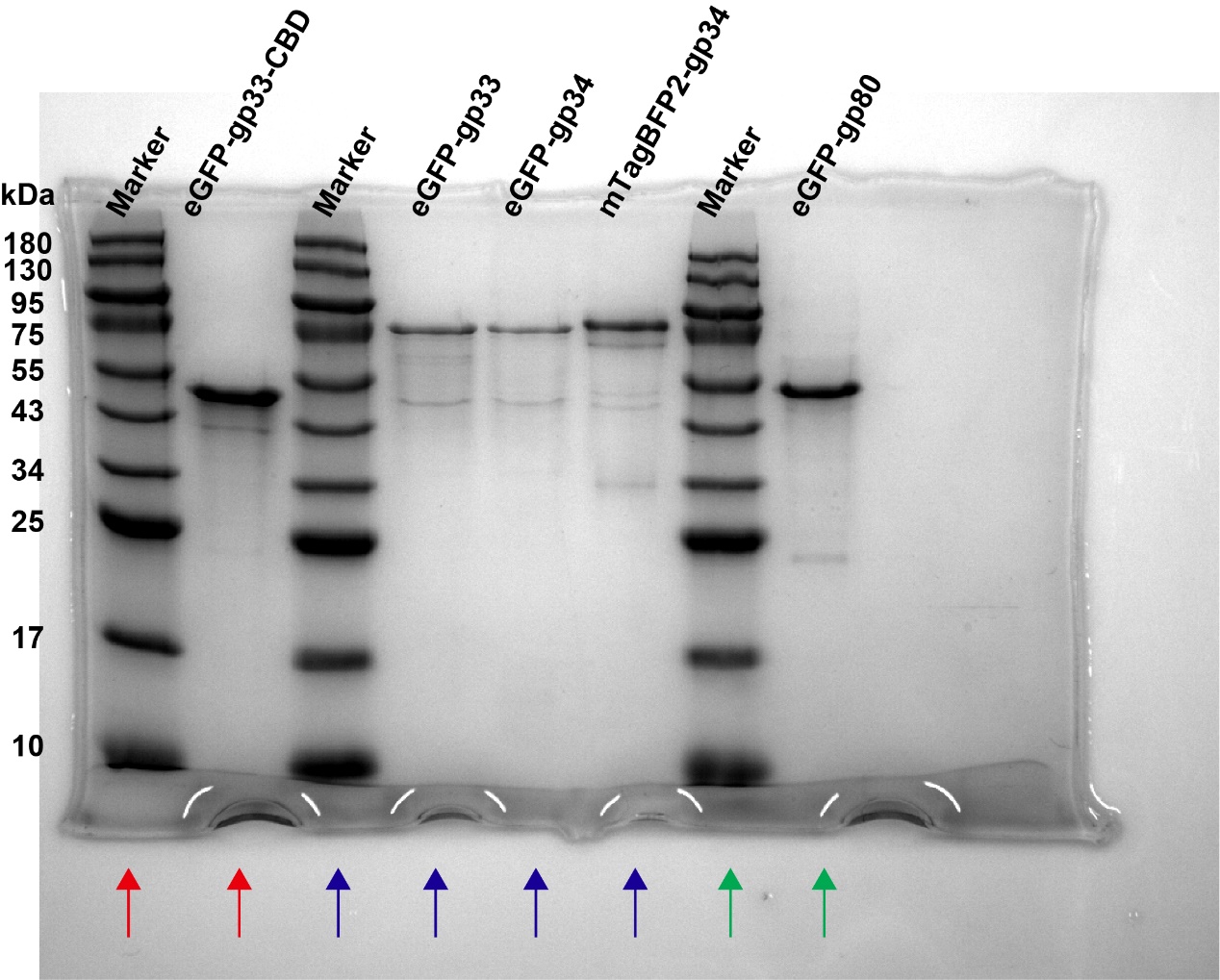


The cropped lanes were indicated by red (S11b), blue (S11c) and green (S11d) arrows, respectively.
